# The Association between Hypertension and Dementia in the Elderly

**DOI:** 10.1155/2012/320648

**Published:** 2011-11-03

**Authors:** Michiya Igase, Katsuhiko Kohara, Tetsuro Miki

**Affiliations:** ^1^Department of Geriatric Medicine, Ehime University Graduate School of Medicine, Toon City, Ehime 791-0295, Japan; ^2^Proteo-Medicine Research Center, Ehime University, Ehime 791-0295, Japan

## Abstract

Hypertension (HT) and dementia are common disorders in the elderly. HT in the elderly is associated with increased occurrence rates of dementia including Alzheimer's disease (AD) and vascular dementia (VaD). In connection to this, some studies have suggested that HT in old age correlates with the pathogenesis of dementia. Since HT is potentially reversible, a number of randomized trials have examined whether antihypertensive treatment may help in preventing dementia occurrence. We review five studies, all using subjects 60 years or older, which investigated different antihypertensive pharmacological treatments. Data from two trials (Syst-Eur, PROGRESS) open the way toward the prevention of dementia (AD or VaD) by antihypertensive treatments. In the Syst-Eur study, with the dihydropyridine calcium antagonists, a reduction in both types of dementia was demonstrated (risk reduction 55%). The PROGRESS study showed that the use of angiotensin-converting enzyme inhibitors (ACEIs), with or without diuretics, resulted in decrease incidence of stroke-related dementia (risk reduction 19%), but dementia without stroke was not reduced. In contrast, the SHEP trial, treatment with a chlorthalidone-based antihypertensive regimen, did not significantly reduced the incidence of dementia. The SCOPE study (candesartan or hydrochlorothiazide versus placebo) and the HYVET-COG study (indapamide or perindopril versus placebo) found no significant difference between the active treatment and placebo group on the incidence of dementia. We found conflicting results regarding treatment benefits in dementia prevention. Recent clinical trials and studies on animal models suggest that blockades of RAS system could have reduced cognitive decline seen in Alzheimer's disease and vascular dementia. Future trials primarily designed to investigate the effects of antihypertensive agents on impaired cognition are needed.

## 1. Introduction

In general, the risk of HT, which is defined as a systolic blood pressure (SBP) ≥140 mm Hg and/or a diastolic blood pressure (DBP) ≥90 mm Hg [1], increases with advancing age. In fact, the prevalence of HT in individuals 60 years and older is double that of those aged 49–59 years. In Framingham study, 90% of all 65-year-old men and women with normal BP later developed HT [2]. This condition carries a very high risk for cerebrovascular disease (CVD) as well as coronary heart disease (CHD) [3]. Dementia is one of the most important neurological disorders in the elderly. Many studies have identified HT as marker for the pathogenesis of dementia AD and VaD, while longitudinal studies have suggested that HT is associated with a higher incidence of dementia in old age. It has been observed that long-standing HT may lead to severe atherosclerosis and impaired cerebrovascular autoregulation, which in turn is thought to correlate with dementia [4]. For these reasons, several studies have investigated whether antihypertensive treatment may retard cognitive decline or dementia [5–9]. Although the importance of lowering BP in HT subjects is well known, the relationship between HT and cognitive function is controversial.

## 2. HT in the Elderly and the Risk of Dementia 

To this date, the associations between BP and dementia have been inconclusive. Considering that the incidence of dementia among the elderly population is rising rapidly worldwide [10] and accumulating evidence that HT may contribute to the development of both AD and VaD [11], there is a reason to believe effective management of HT may translate into major health benefits through the protection of dementia. HT has long been known to cause CV [12]. Midlife HT ranks as an important modifiable risk factor for late-life cognitive decline [13], mild cognitive impairment (MCI) [14, 15], and VaD [16, 17]. In longitudinal cohort studies, elevated BP is associated with cognitive decline although some cross-sectional studies showed mixed relationships between higher BP and cognition, with many studies showing no correlation or even J- or U-shaped associations [18]. Findings from these prospective cohort studies for DBP and cognitive decline are less consistent; however, many have reported a similar inverse relation. The data on the role of BP and HT in later life are not consistent, leaving open the issue of BP treatment in elderly people. The controversy about the association between HT in the elderly and dementia arises because the longitudinal relationship between BP and cognitive change is sensitive to the effects of age, duration of followup and hypertensive treatment status, comorbidity with CVD and CHD, and possibly subclinical dementia [19]. More recently, a total of 668 community-dwelling Japanese individuals without dementia, aged 65 to 79 years, were followed up for 17 years, and examined the associations of late-life and midlife HT with the risk of AD and VaD [20]. During the followup, 123 developed AD, and 76 subjects experienced VaD, and the age- and sex-adjusted incidence of VaD significantly increased with elevated midlife BP levels regardless of late-life BP levels. There were not a significant association between BP levels and AD. Li et al. [21] followed a total of 837 subjects with MCI for 5 years, 298 subjects converted to AD, while 352 remained MCI at the end of the followup. Subjects with HT increased the risk of dementia conversion. Given their results, treatment of HT was associated with a reduced progression in MCI to AD dementia. 

## 3. Can Control of HT Protect against Dementia?

Despite the speculated relationship between HT and dementia, clinical trials examining the preventive effects of antihypertensive therapy on dementia have been inconclusive (Table 1). Among five randomized double-blind placebo-controlled trials surveying antihypertensive treatments and dementia, four (Syst-Eur, PROGRESS, SCOPE, and HYVET-COG) used the Mini-Mental State Examination (MMSE), a widely used screening instrument for cognitive impairment, to assess cognitive function. In the SHEP, cognitive screening was performed by a short-comprehensive assessment and referral evaluation (short CARE) questionnaire. These five studies are described below.

### 3.1. Syst-Eur

The systolic hypertension in Europe study (Syst-Eur) investigated whether antihypertensive treatment in elderly patients with isolated systolic hypertension (ISH) led to a significant change in stroke morbidity and mortality. Syst-Eur investigated the effects of a calcium channel blocker (CCB; 10–40 mg/day nitrendipine). If necessary, nitrendipine was combined with an ACEI (5–20 mg/d enalapril maleate) and/or a diuretic (12.5–25 mg/day hydrochlorothiazide). Participants had no dementia and were at least 60 years old. Their SBP at the beginning of the trial was between 160 and 219 mm Hg, and their DBP was below 95 mm Hg. Antihypertensive therapy began immediately after randomization in the active treatment group, but only after termination of the double-blind trial in the control patients. The mean difference in BP between treatment groups and the control was 7.0 mm Hg SBP and 3.2 mm Hg DBP; the rates of dementia for patients in the active treatment groups and the control groups were 3.3 and 7.4 cases per 1.000 patient-years (relative risk reduction: 55%; 95% CI: 24–73%), respectively, which is significant. 

In Syst-Eur, because active treatment using a CCB resulted in a 42% decrease in the primary end point of fatal and nonfatal stroke, only 2418 of the 4695 randomly assigned patients participated in a substudy on dementia. Compared with the control group, the portion of the treatment group that received only CCB (60%) had a significantly reduced risk of dementia (55%). Interestingly, while the total incidence of dementia was 64 cases, 41 of these showed AD. Therefore, Syst-Eur suggests using a CCB to lower BP may protect against dementia, particularly AD, in elderlywith ISH.

### 3.2. PROGRESS

The perindopril protection against recurrent stroke study (PROGRESS) was a trial involving 6105 patients, all with prior stroke or transient ischemic attack. Participants were assigned to either active treatment (perindopril for all participants plus indapamide for those with neither an indication nor a contraindication to a diuretic) or a matching placebo. The mean difference in BP between the two groups was 9.0 mm Hg SBP and 4.0 mm Hg DBP; the rates of dementia patients in the active treatment groups and the control groups were 6.3% and 7.1% (relative risk reduction: 12%; 95% CI: −8 to 28%), respectively, which is insignificant. However, the rates of cognitive decline were 9.1 and 11.0% (risk reduction: 19%; 95% CI: 4–32%), respectively, which is significant.

### 3.3. SHEP

The systolic hypertension in the elderly program (SHEP) study was a trial conducted over an average 5-year follow-up and involved 16 academic clinics. Among the 447.921 candidates aged 60 years and older screened, 4736 (1.06%) were chosen for the study. SBP at baseline ranged from 160 to 219 mm Hg, while DBP was less than 90 mm Hg. Participants were randomized into either an active antihypertensive drug therapy or a matching placebo group. Active treatment consisted of a diuretic (12.5–25 mg/day chlorthalidone) for step 1 and a beta blockade (25–50 mg/day atenolol) for step 2. If atenolol was contraindicated, 0.05 to 0.10 mg reserpine was used instead. The cohort mean difference in BP between the treatment groups and placebo was 12.0 mm Hg SBP and 4.0 mm Hg DBP; the rates of dementia for the active treatment group and the control group were 3.6 and 4.2 cases per 1.000 patient-years (relative risk reduction: 14%; 95% CI: −26 to 54%), respectively, which is insignificant.

### 3.4. SCOPE

The study on cognition and prognosis in the elderly (SCOPE) was a prospective study conducted from 1997 to 2002. The study involved 4964 patients aged 70–89 years with SBP ranging from 160 to 179 mm Hg and/or DBP ranging from 90 to 99 mm Hg. Patients were assigned randomly to receive the angiotensin II receptor blocker (ARB) candesartan or a placebo, with open-label active antihypertensive therapy added as (84% of patients in the control group). The mean difference in blood pressure between the treatment group and control group was 3.2 mm Hg SBP and 1.6 mm Hg DBP; the rates of dementia for the active treatment group and the control group were 6.3 and 6.8 cases per 1.000 patient-years, respectively, which is insignificant. In a subgroup analysis of SCOPE performed later, a significant positive effect on some cognitive domains (attention and episodic memory) was reported when using testing methods more sensitive than the MMSE.

### 3.5. HYVET-COG

The Hypertension in the very elderly trial—cognitive function assessment (HYVET-COG) examined antihypertensive medication for patients ≥80 years of age. Eligible patients had no dementia, their SBP at entry was 160 to 200 mm Hg, and their DBP was below 110 mm Hg. Participants were randomly assigned to receive 1.5 mg slow release diuretic (indapamide) with the option of ACEI (2–4 mg/day, perindopril), or a placebo. The target SBP was 150/80 mm Hg. Possible cases of dementia (a fall in the MMSE score to <24 or a drop of three points in one year) were assessed by standard diagnostic criteria and expert review. HYVET-COG was the first randomized control study to report the effects of antihypertensive treatment in participants aged 80 years of age or older, finding a significant decrease in stroke after an average followup of 2.2 years, which led to its early termination. This followup period may be too short to detect any benefit preventing dementia. The mean difference in BP between the treatment and control groups was 15 mm Hg SBP and 5.9 mm Hg DBP; the rates of dementia for active treatment group and the control group were 33 and 38 cases per 1.000 patient-years (hazard ratio 0.86; 95% CI: 0.67–1.09), respectively, which is insignificant.

Of these five studies, only Syst-Eur and PROGRESS showed significant differences in the rate of dementia between the treatment and control groups.

However, when four of these data (Syst-Eur, PROGRESS, SHEP, and HYVET-COG) were combined in a meta-analysis [22], antihypertensive therapy was found to significantly reduce the risk of dementia (HR 0.87, 95% CI: 0.76–1.00, *P* = 0.045). Another study, however, found that combining the antihypertensive therapy results from Syst-Eur, SHEP, and SCOPE reduced the risk of dementia by 11% (odds ratio 0.89; 95% CI: 0.69–1.16), an insignificant effect [23]. Therefore, further long-term randomized trials, designed especially to assess a link between antihypertensive therapy and cognition as the primary outcome, are needed.

## 4. Renin-Angiotensin-Aldosterone System and Cognitive Function

Blocking the renin-angiotensin-aldosterone system (RAS) is another means that could have benefits on the prevention of dementia [24], but in a manner independent of BP lowering effect [25]. A potential neuroprotective effect on focal cerebral ischemia has been reported by blocking the RAS with an ARB that specifically targets the angiotensin II receptor [26]. Moreover, the Fournier hypothesis proposes that ARB treatment has potential advantages over ACEI treatment in the prevention of stroke and cognitive impairment because of the lower likelihood of harmful effects like vasoconstriction and proatherothrombogenesis while at the same time promoting neutral or even potentially beneficial effects like vasodilatation and endothelial modulation [27, 28]. 

Consistent with this theory, the ongoing telmisartan alone and in combination with ramipril global endpoint trial (ONTARGET) [29] and the parallel telmisartan randomized assessment study in ACEI intolerant subjects with cardiovascular disease (TRANSCEND) trial [30] have reported the effects of telmisartan, a unique ARB with peroxisome proliferator-activated receptor-gamma (PPAR-gamma-) stimulating activity, and the ACEI ramipril on cognitive function in patients aged 55 years and older with established atherosclerotic cardiovascular disease or diabetes with end-organ damage. In ONTARGET, a 56 month median duration month followup found cognitive impairment occurred in 652 (8%) of the 7865 patients allocated ramipril, 584 (7%) of the 7797 allocated telmisartan, and 618 (8%) of the 7807 allocated a combination of the two (combination versus ramipril, odds ratio [OR] 0.95, 95% CI 0.85–1.07, *P* = 0.39; telmisartan versus ramipril, OR 0.90, 0.80–1.01, *P* = 0.06). Corresponding figures for cognitive decline were 1314 (17%), 1279 (17%), and 1240 (17%), respectively (telmisartan versus ramipril, OR 0.97, 0.89–1.06, *P* = 0.53; combination versus ramipril, OR 0.95, 0.88–1.04, *P* = 0.28). In TRANSCEND, cognitive impairment occurred in 239 (9%) of the 2694 participants allocated telmisartan compared with 245 (9%) of the 2689 allocated a placebo (OR 0.97, 0.81–1.17, *P* = 0.76). The corresponding figures for cognitive decline were 454 (17%) and 412 (16%; OR 1.10, 0.95–1.27, *P* = 0.22), respectively. 

Recently, the prevention regimen for effectively avoiding second strokes (PRoFESS) trial [31] investigated the impact of ARBs on cognitive function in a randomized controlled design. There were no significant differences in the rate of cognitive decline or dementia between the treatment and control groups. These results were very similar to those in SCOPE. However, PRoFESS several limitations that obfuscate its conclusions. For example, the duration of the follow-up period was short, and there was a frequent discontinuation of the study drug among subjects, and many patients experienced recurrent stoke requiring termination of the antihypertensive treatments. 

Figaro et al. [32] reported that antihypertensive therapy with an ARB and diuretic (telmisartan and hydrochlorothiazide) caused significant improvement in cognitive function compared to therapy using an ACEI and diuretic (lisinopril and hydrochlorothiazide). In addition, the observational study on cognitive function and systolic blood pressure reduction (OSCAR), an open label trial in 28 countries designed to evaluate the impact of the ARB eprosartan on cognitive function, found that a reduction in systolic blood pressure had an independent negative association with cognitive decline (odds ratio 0.77; 95% CI: 0.73–0.82). 

More recently, prospective cohort study of old (over 65) subjects demonstrated significantly lower hazard rates for incident dementia with ARBs than with an ACEI (hazard rate 0.81, 95% CI 0.73–0.90) and other cardiovascular drugs (0.76, 0.69–0.84). However, the results may not be generalisable to women because women comprised only 2% of this cohort [33].

Although it is still not exactly clear how the ARBs confer this benefit, Tsukuda et al. [34] demonstrated that a low dose of telmisartan had a preventive effect on cognitive decline in an AD mouse model (A*β*-injection mouse model). This was in part due to the clearance of A*β* in response to an inhibition of inflammation because of PPAR-gamma activation. Thus, ARBs that can act as a partial agonist for PPAR-gamma may provide a benefit for the treatment of dementia, along with their already blood pressure-lowering effects.

## 5. Conclusion

It is thought that there is a dependent relationship between the occurrence of HT and the risk of developing dementia in old age. This offers promise in the prevention of dementia because HT is a potentially reversible risk factor. 

Recent epidemiological evidence suggests that some antihypertensive medications may reduce the risk for AD. In particular, given that Syst-Eur found treating HT with the dihydropyridine CCB nitrendipine reduced the incidence of AD by 55%, nitrendipine could prove to be a potentially reliable option for protection against dementia. Despite this encouraging evidence, several randomized trials have failed to support the efficacy of antihypertensive agents in AD dementia [7, 8]. Thus, at present there is inconsistent evidence regarding the influence of antihypertensive drugs on dementia incidence and/or pathogenesis.

Because current theories assume AD is triggered by the accumulation of soluble and insoluble forms of *β*-amyloid, if high BP can increase the risk of AD, they should then also lead to an accumulation of *β*-amyloid. In vitro study, using primary cortico-hippocampal neuron cultures generated from AD mouse model, at least 7 candidate antihypertensive agents including a calcium blocker, a *β*-adrenergic blocker, an *α*-*β* adrenergic blocker, a diuretic, a vasodilator and ARBs, that significantly reduced AD-type *β*-amyloid protein (A*β*) accumulation [35]. It is thought that some of these drugs may have clinical benefits in protecting against progressive A*β*-related memory disturbance in AD.

Therefore, further prospective randomized studies comparing different antihypertensive classes are needed to provide more evidence regarding the effects of antihypertensive drugs on dementia risk and to determine whether certain antihypertensive classes provide greater benefits than others. In particular, whether these agents possess specific neuroprotective properties or increase cerebral perfusion remains to be clarified.

## Figures and Tables

**Table 1:** Randomized controlled trials about antihypertensive treatments and dementia/cognitive decline.

Study setting	Participants and follow up	Treatment	Test	Main results
Systolic hypertension in Europe study (Syst- Eur) [5]	2.418 systolic hypertensives; mean age 70 years, followup 3.9 years	CCB (nitrendipine) with possible addition of ACE-I (enalapril), diuretic (hydrochlorothiazide), or both versus placebo	MMSE	Mean difference in BP between treatment groups and the control was 7.0 mm Hg SBP and 3.2 mm Hg DBP. Rates of dementia for patients in the active treatment groups and the control groups were 3.3 and 7.4 cases per 1.000 patient-years (relative risk reduction: 55%), respectively. Significant.

The perindopril protection against recurrent stroke study (PROGRESS) [6]	6.105 subjects with prior stroke or transient ischemic attack; mean age 64 years, followup 3.9 years	ACE-I (perindopril) with possible addition of diuretic (indapamide) versus placebo	MMSE	Mean difference in BP between treatment groups and the control was 9.0 mm Hg SBP and 4.0 mm Hg DBP. Rates of cognitive decline for patients in the active treatment groups and the control groups were 11.0 and 9.1% (relative risk reduction: 19%), respectively. Significant.

Systolic hypertension in the elderly program (SHEP) [7]	4.736 systolic hypertensives; mean age 72 years, followup 4.5 years	Diuretic (chlorthalidone) with possible addition of *β* blocker (atenolol) or sympathetic nervous blocker (reserpine) versus placebo	Short CARE	Mean difference in BP between treatment groups and the control was 12.0 mm Hg SBP and 4.0 mm Hg DBP. Rates of dementia incidence for patients in the active treatment groups and the control groups were 3.6 and 4.2 cases per 1.000 patient-years (relative risk reduction: 14%), respectively. Not significant.

Study on cognition and prognosis in the elderly (SCOPE) [8]	4.964 hypertensives; SBP160-170/DBP 90–99 mm Hg; aged 70– 89, followup 3.97 years	ARB (candesartan) versus placebo; open-label antihypertensive drugs were added to both groups	MMSE	Mean difference in BP between treatment groups and the control was 3.2 mm Hg SBP and 1.6 mm Hg DBP. Rates of dementia incidence for patients in the active treatment groups and the control groups were 6.3 and 6.8 cases per 1.000 patient-years, respectively. Not Significant.

Hypertension in the very elderly trial cognitive function assessment (HYVET- COG) [9]	3.336 hypertensives; SBP 160–200 and DBP < 110 mm Hg; age ≤80, followup 2.2 years	Diuretic (indapamide) with possible addition of ACE-I (perindopril) versus placebo	MMSE	Mean difference in BP between treatment groups and the control was 15 mm Hg SBP and 5.9 mm Hg DBP. Rates of dementia incidence for patients in the active treatment groups and the control groups were 33 and 38 cases per 1.000 patient-years (hazard ratio 0.86). respectively. Not significant.

BP: blood pressure, SBP: systolic blood pressure, DBP: diastolic blood pressure.
